# Monthly Summary

**Published:** 1891-12

**Authors:** 


### Monthly Summary.

A CASE OF COCAINE POISONING—*Reported by Dr. T. C. Edwards, Gainesville, Texas.*—On September 23rd a lady, the wife of a railroad conductor, called to have the right superior first molar extracted. The crown was gone; roots and socket very sore from peridental inflammation. A 12 per cent. solution of hydrochlorate cocaine was applied to the surface of the gum twice at an interval of five minutes; none used hypodermically. After another interval of five minutes the tooth was extracted, but it required three efforts to get the roots which had to be separated. The operation gave great pain notwithstanding the use of the cocaine. No bad effects were noticed from the cocaine used.

She had an upper left wisdom to be extracted, but postponed it for another sitting. On the 2nd of October, (about a weak after this) she came accompanied by her husband to have the wisdom tooth extracted. She was very nervous and fearful about the expected operation and, in fact, was almost in a state of nervous prostration from worry, pain and loss of sleep· as the tooth had been aching very severely for several days.

This time a 12 per cent. solution of the cocaine was applied to the gums as before, after which a 4 per cent. solution of cocaine was injected first on the outside or buccal surface of the tooth and then on the palatine surface, passing the needle deeply into the tissues alongside the tooth—about one-half inch I think.

Almost immediately after removing the needle—the last injection—symptoms of cocaine poisoning began to show themselves. The first effects noticeable were those of a person just in the act of fainting, followed in quick succession by violent twitching, convulsive movements of the eyeballs, the general tendency of which seemed to be upward, under the upper lids, crossings of the eyes, etc. The respiration became very short and quick as if a paralysis of the lungs were coming on; the pulse became very rapid, weak and thready, in fact, was at times almost imperceptible. One peculiarity of her condition was that for short intervals the violent and alarming symptoms would pass away and she would appear to be reacting, but only momentarily, for she would relapse again into more violent paroxysms than before. During this time she was apparently unconscious. There is reason to believe that she was entirely so.

Of course prompt measures were taken to restore her. The chair was thrown back until her head was brought below a horizontal line. Cold, wet towels applied to her head and face, her clothing loosened and a physician sent for. As soon as it could be procured, which, fortunately, was in a very few minutes, she was given a drink of hot, strong coffee; the effect of which was like magic, for her alarming symptoms subsided at once. Her breathing became easier, her pulses stronger, she became conscious, in fact, in a few minutes she was out of danger. She was very much prostrated being so weak, that it was several hours before she could leave the chair. The physician gave her a dose of digitalis before he left and she was also given brandy afterwards, but the remedy that relieved her was the coffee.

This is the history of the case. Some may say there is nothing serious about that, but if they had seen the case and been responsible for it they would doubtless think differently. She came back and had the tooth extracted two days afterwards without difficulty and without cocaine.—*Texas Dental Journal.*

---

CAPPING PULPS.—It is more amusing than instructive to listen to the discussion in almost any well regulated dental society when the subject of capping pulps arises. The various contradictory methods that are recommended, and the direct conflict of principles involved, are not calculated to impress the man up a tree with an exalted estimate of the scientific status of the disputants. One insists upon the absolute necessity for the use of a cauterant, that the exposed and irritated tissues may be destroyed. Another carefully avoids these agents, because the pellicle thus formed must become a dangerous element. One desires the influence of a moderate irritant, that the pulp may be stimulated to action in depositing secondary dentine, while another insists upon the use of only the very blandest and most soothing applications.

One would place over the exposed point a metallic cap, while another demands a porous covering, that may absorb any effusions from the wounded pulp. One uses a concave disk of some kind, to form a bridge above the breach in the dentinal walls, while another contends for a material that shall be in absolute contact with it.

One will deplete the pulp by bleeding, while another holds up his hands in horror at any willful traumatism. One employs as a covering oxy-phosphate of zinc, because it is a non-irritant; another, oxy-chloride, because it is an irritating stimulant. One uses gutta-percha, because it is a non-conductor; another platinum, because it is not. One applies an obtundent to keep it quiet; another deprecates this, because it induces a fatal sleep. One varnishes the exposed point, another dusts it over with some preparation of lime, while yet another whacks in a filling with scarce any protective measures at all. A dentist once took out a patent for covering the pulp with a piece of quill, and retailed out his goose-feather cappings at an extravagant price.

And what is yet more amusing, each of the advocates of these contradictory methods claims to meet with almost universal success. Each is ready to condemn every other method, but he stands up for his own particular fad as almost infallible. If one-tenth of the pulps are really saved that it is claimed are preserved alive, dental practice must either be an awful humbug, or the tooth-pulp is the most enduring of all the notoriously abused organs of the body.

Amid all this contradiction, the impartial spectator referred to, the man up the tree, looks on and wonders when some settled principle will govern the matter; when some intelligent and consistent line of practice will be adopted; when some physiological, or chemical, or empirical law will be discovered, that will be a rule of procedure; when the present hodgepodge of nonsensical nostrums will be abandoned; when it will be found out whether exposed pulps can or cannot be saved, and what reasonable and really scientific method will supply the greatest chance for a favorable result.—*Dental Pract. and Advertiser.*

---

FITTING BANDS.—Every crown-worker has his own method of fitting a gold band around the root of a tooth, and we do not pretend to be an exception to the rule. The one we are now using is that for which we have abandoned a number of other methods. Perhaps it may not meet the approval of others—and perhaps many others are now using it.

A strip of gold, the proper length and width, is cut and annealed to perfect softness. It is then, with pliers, approximately fitted about the root, and a strong waxed ligature wound about it—usually twice—with a single surgeon's knot placed in it. While the assistant holds the band in place by her finger upon the top of it, the ligature is firmly drawn up The cervical edge of the band is carefully burnished into place, and the overlapping end pressed down. The ligature is again and again drawn up, until the band is perfect in its adaptation. Then it is finally tied, the band slipped off, grasped by a pair of delicate pliers at the lap, a bit of solder placed on the inside, and it is held in the flame until soldered. The ligature is left to burn off in the flame.

The adapting of the cervical edge of the band to the festoon of the gum may be partially done before the ligature is placed about it, or it may be entirely left until after the soldering is done, according to the necessities of the case.

There is nothing better for ligatures than three-cord, linen machine thread, about No. 30, such as is sold for shoemaker's use. It may be obtained at shoemaker's outfitting establishments.—*Dent. Pract. and Advertiser.*

---

THE PHYSIOLOGY OF DIGESTION IN INFANTS.—Dr. Booker found that milk remains in the stomach of infants for a much shorter time than in adults. The period depends upon the character of the milk and the age of the patient. In breast-fed children, during the first week, the stomach is sometimes found empty within an hour after nursing. The maximum period for breast-fed children during the first week is an hour and a half, and two hours for older children and those fed on cows' milk. It has not yet been determined whether the milk passes from the stomach continuously or periodically. During the few minutes after feeding, the reaction of the contents of the stomach is that of the food that was taken. After fifteen minutes, it is always acid. *Milk absorbs hydrochloride acid so rapidly that free acid does not appear until late.*

In disordered conditions of the digestion, milk remains much longer in the stomach than in health. Milk curds are sometimes washed out four or five hours after feeding. They sometimes become very hard and of bad odor. Excessive secretion of mucus usually accompanies disordered digestion. —*Johns Hopkins Bulletin*, July, 1890.

---

GORGAS' DENTAL MEDICINE, Fourth Edition, Revised and Enlarged, is on our table. The rapidity with which this work has attained to the fourth edition is sufficient evidence of its popularity. To quote from the preface to this edition: "So much has been added to this (fourth) edition, in order to bring the work up to the present status of dental materia medica and therapeutics, that the author cherishes the hope that its value as a text book and work of reference has been greatly increased over that of former editions." The new chapter which has been added on the use of antiseptics in dental practice, together with the list of new antiseptics, disinfectants, germicides and hypnotics, contributes to increase the value of the work. It is a useful addition to any dentist's library.

The work is published by P. Blakiston, Son & Co., No. 1012 Walnut street, Philadelphia.—*Texas Dent. Journal.*

---

KEEP CLEAN.—An unkempt, slovenly, dirty dentist is a nuisance. If you can't afford nice professional clothes, shut up shop and earn them at the anvil or in the potato patch. Look neat and clean anyway. Starve yourself if necessary, but look presentable. Keep your mouth clean, too, and your breath. Away with that nasty stuff that so defiles the whole body. The idea of burning it in your mouth and making a chimney of your nose! it is an offence to your best customers. Have a bath tub and get in it often and scrub yourself till you shine. The best cosmetic is thorough rubbing, and the best perfume is cleanliness.—Editorial in *Items of Interest.*

